# Exploring the interaction among EPHX1, GSTP1, SERPINE2, and TGFB1 contributing to the quantitative traits of chronic obstructive pulmonary disease in Chinese Han population

**DOI:** 10.1186/s40246-016-0076-0

**Published:** 2016-05-18

**Authors:** Li An, Yingxiang Lin, Ting Yang, Lin Hua

**Affiliations:** Beijing Key Laboratory of Respiratory and Pulmonary Circulation Disorders, Beijing Institute of Respiratory Medicine, Department of Respiratory and Critical Care Medicine, Beijing Chao-Yang Hospital, Capital Medical University, Beijing, 100020 China; Department of Respiratory and Critical Care Medicine, China-Japan Friendship Hospital, Beijing, 100029 China; School of Biomedical Engineering, Capital Medical University, Beijing, 100069 China; Beijing Key Laboratory of Fundamental Research on Biomechanics in Clinical Application, School of Biomedical Engineering, Capital Medical University, Beijing, 100069 China

**Keywords:** COPD, SNP, Interaction, Quantitative traits, MDR

## Abstract

**Background:**

Currently, the majority of genetic association studies on chronic obstructive pulmonary disease (COPD) risk focused on identifying the individual effects of single nucleotide polymorphisms (SNPs) as well as their interaction effects on the disease. However, conventional genetic studies often use binary disease status as the primary phenotype, but for COPD, many quantitative traits have the potential correlation with the disease status and closely reflect pathological changes.

**Method:**

Here, we genotyped 44 SNPs from four genes (EPHX1, GSTP1, SERPINE2, and TGFB1) in 310 patients and 203 controls which belonged to the Chinese Han population to test the two-way and three-way genetic interactions with COPD-related quantitative traits using recently developed generalized multifactor dimensionality reduction (GMDR) and quantitative multifactor dimensionality reduction (QMDR) algorithms.

**Results:**

Based on the 310 patients and the whole samples of 513 subjects, the best gene-gene interactions models were detected for four lung-function-related quantitative traits. For the forced expiratory volume in 1 s (FEV1), the best interaction was seen from EPHX1, SERPINE2, and GSTP1. For FEV_1_%pre, the forced vital capacity (FVC), and FEV_1_/FVC, the best interactions were seen from SERPINE2 and TGFB1.

**Conclusion:**

The results of this study provide further evidence for the genotype combinations at risk of developing COPD in Chinese Han population and improve the understanding on the genetic etiology of COPD and COPD-related quantitative traits.

**Electronic supplementary material:**

The online version of this article (doi:10.1186/s40246-016-0076-0) contains supplementary material, which is available to authorized users.

## Background

Chronic obstructive pulmonary disease (COPD) is defined as airflow limitation that is not fully reversible [1]. Cigarette smoking is the major risk factor for COPD, but smokers show considerable variation in their risk of developing airflow obstruction [2]. Although a series of studies have found the genetics contributions from some genes by analyzing individual effects of single nucleotide polymorphisms (SNPs) [3], but in most cases with a large proportion of the genetic component left unexplained, the genetic risk factors for COPD are still largely unknown. Recent studies have approved that complex traits cannot be explained by any single SNP variant, and the characterization of gene-gene interactions and gene-environment interactions may be the key to understand the underlying pathogenesis of complex diseases [4]. It is therefore suggested that identifying the possible jointed effects of gene-gene interaction will help discover the potential susceptibility factors of COPD risk.

Recent advances in genetic studies have implicated that COPD represents a complex disease with genetics contributions from multiple genes. For example, although many studies did not find the association between EPHX1 and the susceptibility to COPD or disease severity [5, 6], Vibhuti et al. [7] approved that and the combination of 113H/139H alleles of mEPHX and 105V/114V alleles of GSTP1 genotypes with same alleles is associated with imbalanced oxidative stress and lung function in COPD patients. In addition, Artigas et al. [8] investigated the combined effect of the risk alleles at six loci (TNS1, GSTCD, HTR4, AGER, THSD4, and HHIP) and found their joint effects on lung function and COPD risk. Therefore, as a complex polygenic disease, COPD is likely affected by the operation of multiple genes and the coincident actions of several genetic events. However, most conventional genetic studies often use binary disease status as the primary phenotype, but for COPD, many quantitative traits have been shown to correlate with the disease status and to have greater sensitivity in detecting early pathological changes. For example, a ratio between forced expiratory volume in 1 s (FEV_1_) and forced vital capacity (FVC) has emphasized the importance of the functional assessment of usually progressive, non-fully reversible airflow limitation [9]. The BODE index, a multidimensional parameter including the body-mass index (B), the degree of airflow obstruction (O), functional dyspnea (D), and exercise capacity (E), was reported to be superior to FEV_1_ in reflecting the severity of COPD and effective in predicting the mortality in patients with COPD [10]. Therefore, it is suggested that important insight can be garnered from investigating genotype combinations contributing to the COPD-related quantitative traits, which will help improve the understanding of the genetic etiology of COPD.

Recently, some extension algorithms for detecting and characterizing epistatic interactions in the context of quantitative outcomes named as generalized multifactor dimensionality reduction (GMDR) [11] and quantitative multifactor dimensionality reduction (QMDR) were developed [12], and these algorithms allow researchers to build more accurate models that involve multiple genotype combinations contributing to disease-related quantitative traits. By summarizing a group/body of evidence, we found four genes: EPHX1, GSTP1, SERPINE2, and TGFB1, that are reported to be associated with COPD, and these associations have been confirmed by replication and meta-analysis [13]. Therefore, in the present study, we genotyped 44 SNPs (Additional file 1) involved in these four genes in 310 COPD patients and 203 controls which belonged to the Chinese Han population to explore the joint gene-gene interactions contributing to COPD-related quantitative traits based on the GMDR and QMDR algorithms. We also used a web-based tool GeneMANIA [14] to find genes that interact in any way (physically, genetically, etc.) with our studied four genes in this paper. Our study provides further evidences to identify genotype combinations at risk of developing COPD in Chinese Han population and improve the understanding of the genetic etiology of COPD.

## Results

### General characteristics of COPD-related quantitative traits for patients

The general characteristics of COPD-related quantitative traits for patients are shown in Additional file 2. Especially, the distribution of the modified Medical Research Council dyspnea scale (MMRC) is as follows: 52 patients (16.8 %) MMRC 0, 144 (46.5 %) MMRC 1, 84 (27.1 %) MMRC 2, 11 (3.5 %) MMRC 3, and 19 (6.1 %) MMRC 4, with a median of 1 (*P*_5_–*P*_95_, 0–4). This MMRC distribution indicates that the degrees of various physical activities that precipitate dyspnea of the COPD patients in our study are mild. The distribution of BODE index is as follows: 9 patients (2.9 %) BODE 0, 56 (18.1 %) BODE 1, 93 (30.0 %) BODE 2, 71 (22.9 %) BODE 3, 36 (11.6 %) BODE 4, 26 (8.4 %) BODE 5, 11 (3.5 %) BODE 6, 4 (1.3 %) BODE 7, 3 (1.0 %) BODE 9, and 1 (0.3 %) BODE 10, with a median of 2 (*P*_5_–*P*_95_, 1–6). The BODE distribution also indicates that the severity of our COPD patients is mild. In addition, in terms of the effect of a single SNP, except rs41266229 (EPHX1) and rs729631 (SERPINE2) which display the significant difference between the COPD patients and normal controls after passing the multiple testing corrections [13], no significant differences were seen from other single SNP (Additional file 1).

### Single marker analysis for COPD-related quantitative traits based on patients

In the current study, we performed the analysis of single marker effect for seven COPD-related quantitative traits. We included covariates age, sex, and pack-years of smoking in the model. For the seven COPD-related quantitative traits, except rs729631 (SERPINE2) which showed a strong association with FVC, no single marker effects showed a significant association after being corrected by Bonferroni procedure (Table 1). It appears that most of these candidate SNPs do not show independent associations with COPD-related quantitative traits.

**Table 1:** Single marker effects for six^a^ COPD-related quantitative traits (*n* = 310 patients)

Six COPD-related quantitative traits																	
FEV_1_			FEV_1_%pre			FVC			FEV_1_/FVC (%)			BODE			6MWT		
Main effects	p*	p**	Main effects	p*	p**	Main effects	p*	p**	Main effects	p*	p*	Main effects	p*	p**	Main effects	p*	p**
SERPINE2 (rs729631)	0.004	0.176	EPHX1 (rs868966)	0.048	1.000	EPHX1 (rs868966)	0.010	0.440	EPHX1 (rs3738040)	0.005	0.220	SERPINE2 (rs4674841)	0.008	0.352	EPHX1 (rs3766934)	0.027	1.000
SERPINE2 (rs975278)	0.016	0.704				EPHX1 (rs2854450)	0.022	0.968	EPHX1 (rs1877724)	0.007	0.308	EPHX1 (rs2234922)	0.019	0.836	SERPINE2 (rs4674841)	0.045	1.000
SERPINE2 (rs4674841)	0.037	1.000				EPHX1 (rs3738040)	0.023	1.000	EPHX1 (rs2292558)	0.015	0.660	GSTP1 (rs1138272)	0.020	0.880	SERPINE2 (rs6734100)	0.046	1.000
SERPINE2 (rs17196253)	0.013	0.572				EPHX1 (rs1877724)	0.045	1.000	GSTP1 (rs1138272)	0.028	1.000	SERPINE2 (rs13392495)	0.025	1.000			
SERPINE2 (rs3820766)	0.029	1.000				SERPINE2 (rs729631)	2.27E-05	0.001	SERPINE2 (rs729631)	0.002	0.088	SERPINE2 (rs2118409)	0.032	1.000			
									SERPINE2 (rs975278)	0.035	1.000	GSTP1 (rs36211088)	0.032	1.000			

^a^There was no significant main effect for MMRC before being corrected by Bonferroni procedure

*p < 0.05 before corrected by Bonferroni procedure; **p after corrected by Bonferroni procedure

### The best two-way gene-gene interaction models for COPD-related quantitative traits using GMDR, QMDR, and traditional quantitative trait locus (QTL)

**Fig. 1:**
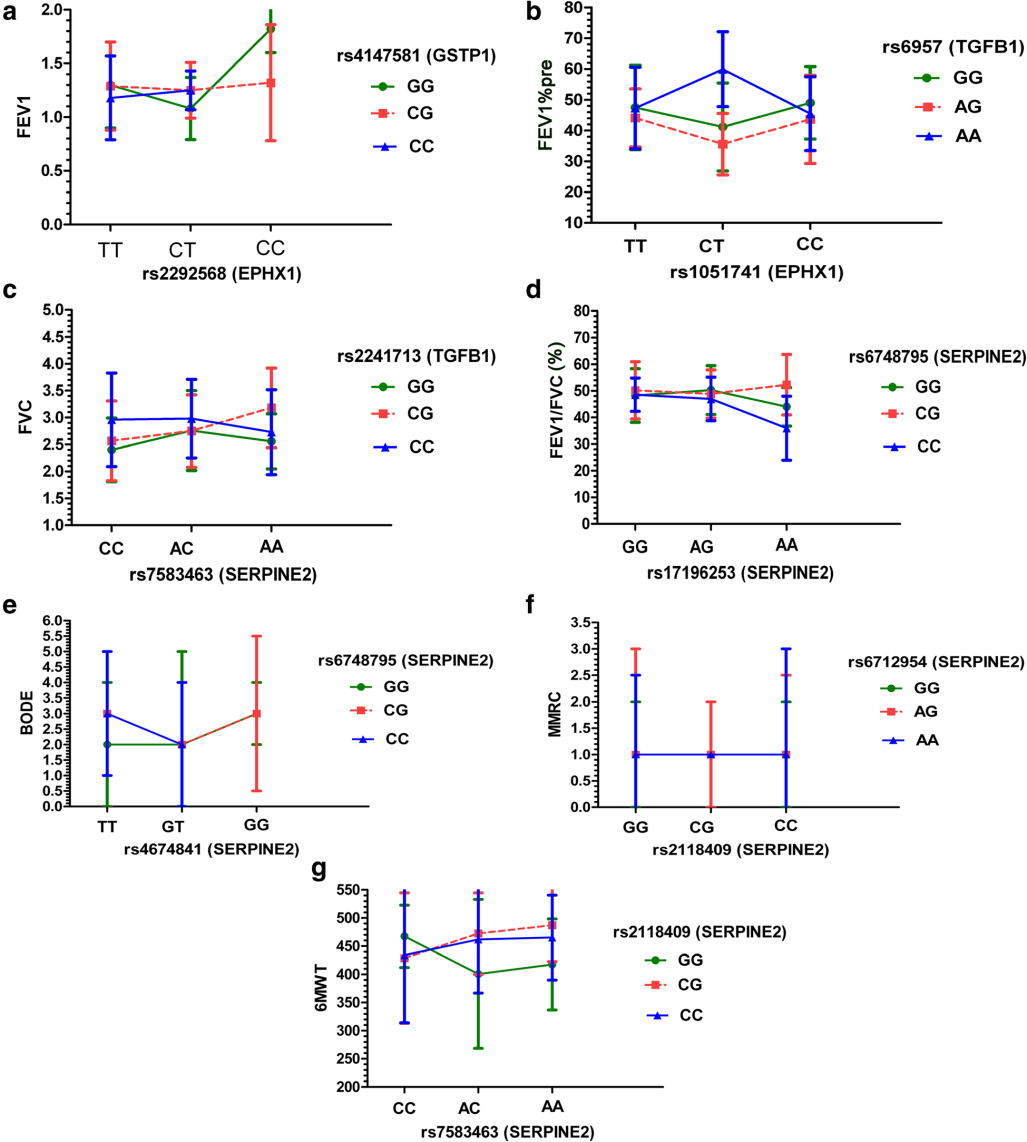
The best gene-gene interaction models for COPD-related quantitative traits using QMDR method. **a** FEV_1_. **b** FEV_1_%pre. **c** FVC. **d** FEV_1_/FVC (%). **e** BODE. **f** MMRC. **g** 6MWT. For FEV_1_, FEV_1_%pre, FVC, FEV_1_/FVC, and 6MWT, the *y-axis* represents the mean of the trait and the *error bar* represents the standard deviation. For BODE and MMRC, the *y-axis* represents the median of the trait and the *error bar* represents the quartile interval

In Table 2, we listed the best interaction models identified by QMDR from tenfold cross-validation for COPD-related quantitative traits after being adjusted by covariates age, sex, and pack-years of smoking based on 310 patients. For FEV_1_, the best model is the interaction between EPHX1 (rs2292568) and GSTP1 (rs4147581). The 1000 permutation testing revealed a significant *P* value of 0.019. The average maximum of FEV_1_ (1.82) was seen from rs2292568 (CC)*rs4147581 (GG), whereas the average minimum of FEV_1_ (1.08) was seen from rs2292568 (CT)*rs4147581 (GG) (Fig. 1a). This indicated that having two minor alleles for the EPHX1 gene corresponded to an average maximum value of FEV_1_. For FEV_1_%pre, the best interaction was detected between rs1051741 (EPHX1) and rs6957 (TGFB1). The 1000 permutation testing also revealed a significant *P* value of 0.042. The average maximum of FEV_1_%pre (59.92) was seen from rs1051741 (CT)*rs6957 (AA), whereas the average minimum of FEV_1_%pre (35.57) was seen from rs1051741 (CT)*rs6957 (AG) (Fig. 1b). For FVC, the best model is the interaction between SERPINE2 (rs7583463) and TGFB1 (rs2241713) which shows a significant *P* value of 0.028 based on the 1000 permutation testing. The average maximum of FVC (3.18) was seen from rs7583463 (AA)*rs2241713 (CG), whereas the average minimum of FVC (2.40) was seen from rs7583463 (CC)*rs2241713 (GG) (Fig. 1c). However, for the other four COPD-related quantitative traits, the best interaction models were found within the gene itself but not found between genes, and the 1000 permutation testing did not find their significance (*P* > 0.05). For FEV_1_/FVC, 6-min walk test (6MWT), BODE, and MMRC, the best interactions are rs17196253 (SERPINE2) and rs6748795 (SERPINE2) (Fig. 1d), rs7583463 (SERPINE2) and rs2118409 (SERPINE2) (Fig. 1e), rs4674841 (SERPINE2) and rs6748795 (SERPINE2) (Fig. 1f), and rs2118409 (SERPINE2) and rs6712954 (SERPINE2) (Fig. 1g), respectively. The description and comparison among multiple genotype combinations contributing to seven COPD-related quantitative traits are shown in Additional file 3. For the 310 patients, after being adjusted by covariates, the most significant gene-gene interactions identified by traditional QTL were consistent with those interactions obtained from QMDR in five quantitative traits: FEV_1_, FEV_1_%pre, FVC, FEV_1_/FVC, and BODE (Additional files 4 and 5). For MMRC and 6MWT, there were some discrepancies between QMDR and traditional QTL. We know that QTL is less sensitive in detecting the local effects, whereas QMDR can find the genotype combinations that distinguish the high- and low-level groups optimally. Therefore, when there is good discrimination between the low- and high-level groups in terms of traits, these two methods can both detect the interactions with good power.

**Table 2:** The best models identified by QMDR for COPD-related quantitative traits in Chinese Han population (*n* = 310 patients)

COPD-related quantitative traits	The best two-way interaction models	T-CV score	CV consistency	Permutated *P* value
FEV_1_	EPHX1(rs2292568)*GSTP1(rs4147581)	2.6390	7/10	0.019*
FEV_1_%pre	EPHX1(rs1051741)*TGFB1(rs6957)	1.9234	7/10	0.042*
FVC	SERPINE2(rs7583463)*TGFB1(rs2241713)	2.2735	8/10	0.028*
FEV_1_/FVC (%)	SERPINE2(rs17196253)*SERPINE2(rs6748795)	0.3566	3/10	NS
BODE	SERPINE2(rs4674841)*SERPINE2 (rs6748795)	−0.3295	4/10	NS
MMRC	SERPINE2(rs2118409)*SERPINE2 (rs6712954)	1.467	2/10	NS
6MWT	SERPINE2(rs7583463)*SERPINE2 (rs2118409)	1.229	7/10	NS

*CV* cross-validation, *NS* not significant

*P value obtained from the 1000 permutation testing

In addition, for lung-function-related quantitative traits (FEV_1_, FEV_1_%pre, FVC, and FEV_1_/FVC), we further detected the two-way gene-gene interaction using GMDR, QMDR, and traditional QTL based on the whole samples (310 patients and 203 controls). GMDR, QMDR, and traditional QTL all found that the best interaction model was EPHX1 and GSTP1 for FEV_1_, SERPINE2 and TGFB1 for FVC, and FEV_1_/FVC (Table 3 and Additional file 4).

**Table 3:** The best two-way models identified by QMDR and GMDR for four lung-function-related quantitative traits in Chinese Han population (*n* = 310 patients + 203 controls)

COPD-related quantitative traits	The best two-way interaction models based on QMDR			The best two-way interaction models based on GMDR		
	Gene-gene interaction	CV consistency	Permutated *P* value	Gene-gene interaction	CV consistency	Permutated *P* value
FEV_1_	rs2260863(EPHX1)*rs4147581(GSTP1)	5/10	0.031*	rs3766934(EPHX1)*rs947895(GSTP1)	3/10	NS
FEV_1_%pre	rs10151740(EPHX1)*rs1800469(TGFB1)	2/10	NS	rs2260863(EPHX1)*rs861442(SERPINE2)	3/10	NS
FVC	rs7583463(SERPINE2)*rs2241713(TGFB1)	5/10	NS	rs6748795(SERPINE2)*rs2241713(TGFB1)	4/10	0.036*
FEV_1_/FVC (%)	rs17196253(SERPINE2)*rs2241713(TGFB1)	8/10	0.027*	rs729631(SERPINE2)*rs2241713(TGFB1)	2/10	NS

*CV* cross-validation, *NS* not significant

*P value obtained from the 1000 permutation testing

### The best three-way gene-gene interactions models for COPD-related quantitative traits using GMDR and QMDR

In Table 4, we listed the best three-way gene-gene interaction models identified by QMDR from tenfold cross-validation for COPD-related quantitative traits after being adjusted by covariates age, sex, and pack-years of smoking based on 310 patients. For FEV_1_, the best model was seen from GSTP1, EPHX1, and SERPINE2. For FEV_1_%pre, FVC, and FEV_1_/FVC, the best models were seen from SERPINE2 and TGFB1. For BODE, EPHX1 and SERPINE2 were found to be the best interaction. For MMRC, interaction between GSTP1, TGFB1, and SERPINE2 was the best model with a significant permutated *P* value of 0.022. For 6MWT, the best model was SERPINE2 and GSTP1.

**Table 4:** The best three-way models identified by QMDR for COPD-related quantitative traits in Chinese Han population (*n* = 310 patients)

COPD-related quantitative traits	The best three-way interaction models	T-CV score	CV consistency	Permutated *P* value
FEV_1_	rs4147581(GSTP1)*rs2292568(EPHX1)*rs4674843(SERPINE2)	0.9948	3/10	NS
FEV_1_%pre	rs282254(SERPINE2)*rs6738983(SERPINE2)*rs2241713(TGFB1)	0.4451	3/10	NS
FVC	rs2241715(TGFB1)*rs282254(SERPINE2)*rs6738983(SERPINE2)	−0.2469	2/10	NS
FEV_1_/FVC (%)	rs10191694(SERPINE2)*rs282254(SERPINE2)*rs2241713(TGFB1)	0.0393	4/10	NS
BODE	rs13392495(SERPINE2)*rs2118409(SERPINE2)*rs2234922(EPHX1)	−0.1974	2/10	NS
MMRC	rs4147581(GSTP1)*rs2241718(TGFB1)*rs2118409(SERPINE2)	−2.3572	2/10	0.022*
6MWT	rs4674841(SERPINE2)*rs7583463(SERPINE2)*rs947895(GSTP1)	0.9901	2/10	NS

*CV* cross-validation, *NS* not significant

*P value obtained from the 1000 permutation testing

In addition, for four lung-function-related quantitative traits, we also detected the three-way gene-gene interactions using GMDR and QMDR based on the whole samples. GMDR and QMDR all found that the best interaction model was EPHX1, GSTP1, and SERPINE2 for FEV_1_ and SERPINE2 and TGFB1 for FVC (Table 5).

**Table 5:** The best three-way models identified by QMDR and GMDR for four lung-function-related quantitative traits in Chinese Han population (*n* = 310 patients + 203 controls)

COPD-related quantitative traits	The best three-way interaction models based on QMDR			The best three-way interaction models based on GMDR		
	Gene-gene interactions	CV consistency	Permutated *P* value	Gene-gene interactions	CV consistency	Permutated *P* value
FEV_1_	rs4147581(GSTP1)*rs2260863(EPHX1)*rs6736436(SERPINE2)	4/10	NS	rs868966(EPHX1)*rs7583463(SERPINE2)*rs947895(GSTP1)	4/10	NS
FEV_1_%pre	rs282254(SERPINE2)*rs6738983(SERPINE2)*rs2241713(TGFB1)	4/10	0.041*	rs282254(SERPINE2)*rs729631(SERPINE2)*rs1051740(EPHX1)	2/10	NS
FVC	rs2241715(TGFB1)*rs282254(SERPINE2)*rs6738983(SERPINE2)	2/10	NS	rs2241713(TGFB1)*rs6748795(SERPINE2)*rs7579646(SERPINE2)	4/10	0.035*
FEV_1_/FVC (%)	rs10191694(SERPINE2)*rs868966(EPHX1)*rs2241713(TGFB1)	4/10	NS	rs4674843(SERPINE2)*rs282254(SERPINE2)*rs2241713(TGFB1)	5/10	0.039*

*CV* cross-validation, *NS* not significant

^*^ *P* value obtained from the 1000 permutation testing

### Gene-gene interactions in the network

**Fig. 2:**
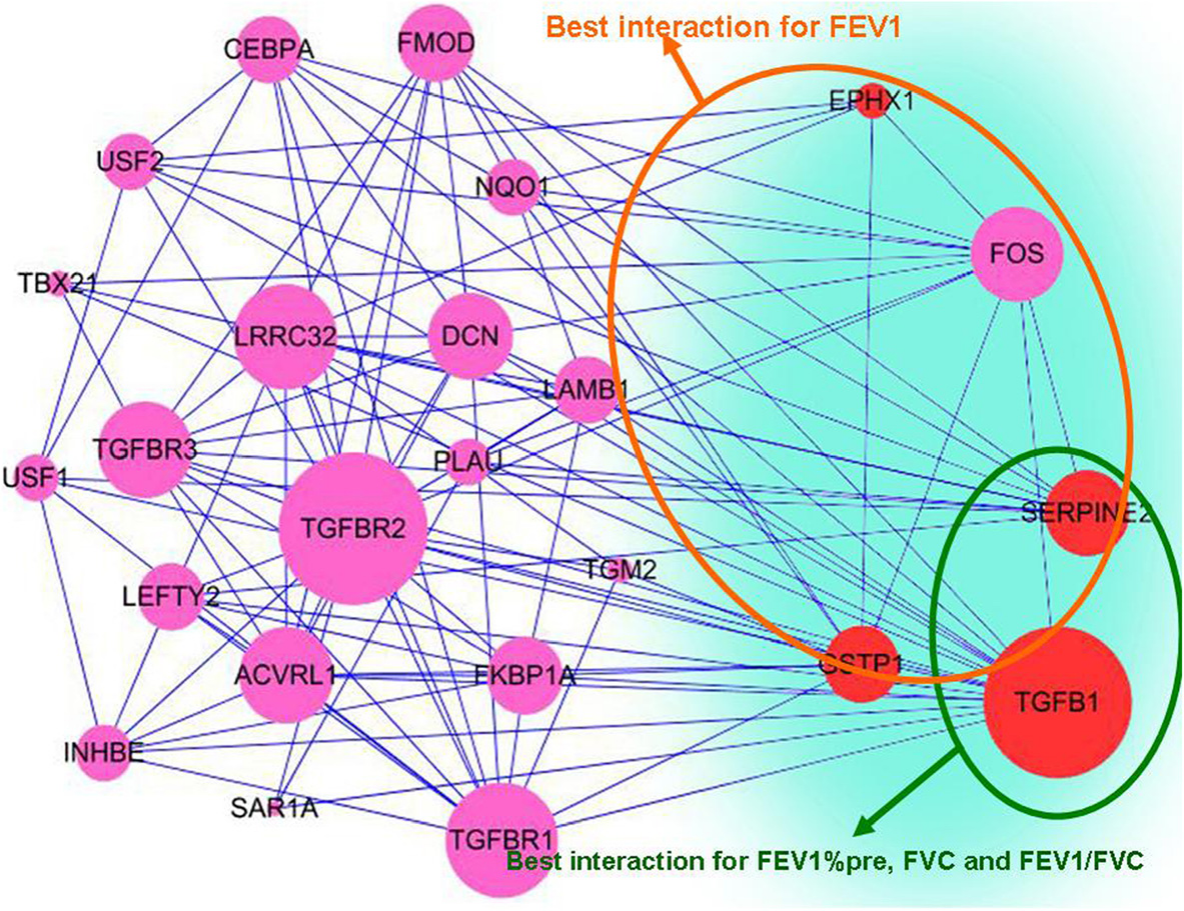
The interaction of four genes in the network using GeneMANIA web tool. The *red circles* indicate the four genes studied in this paper, and other *pink circles* indicate the interacted genes in the network acquired from GeneMANIA web tool. The *larger size circles* indicate the genes with the greater degree in the network

To extend/explore the potential joint genetic effects of these four genes (EPHX1, GSTP1, SERPINE2, and TGFB1), we also used a web-based tool GeneMANIA [14] to find their interactions in any way (physically, genetically, etc.) in the network. We found that except EPHX1 which interacted with GSTP1 directly in the network, these four genes interacted with each other indirectly by passing a transcription factor named FOS (Fig. 2). Previous study confirmed the altered expression of gene encode for FOS in the lung tissues from COPD by using real-time quantitative RT-PCR [15]. However, Demoly et al. found that FOS was rarely expressed in the normal and pathological chronic bronchitis and lung cancer proliferative compartment of the human bronchi, suggesting its low role in cell proliferation of the large airways [16]. Considering that FOS is the center of the interaction network involved in these four genes, it should be focused on in further studies.

## Discussion

Up to now, although there are many different candidate genes which have been investigated for their potential roles in lung function impairment in smokers [17, 18], few works were interested to study the combinations of polymorphisms in COPD quantitative traits. In this paper, our study tested for the association of genetic interaction with seven COPD-related quantitative traits using recently developed GMDR and QMDR algorithms. We got the support for the lack of single marker associations between these SNPs and COPD-related quantitative traits; however, our quantitative trait interaction analysis yielded several interesting candidate gene-gene interactions. For FEV_1_, the best interaction was seen from EPHX1, SERPINE2, and GSTP1. For FEV_1_%pre, FVC, and FEV_1_/FVC, the best interaction models were seen from SERPINE2 and TGFB1. Interestingly, we found EPHX1 interact with GSTP1 directly for FEV_1_ trait for COPD patients, which is consistent with the interaction identified by GeneMANIA. In previous studies, Lakhdara et al. have suggested that combined EPHX1, GSTP1, GSTM1 and GSTT1 genetic polymorphisms may play a significant role in the development of COPD, emphysema and decline of the lung function based on the analysis for Tunisian population [18]. Salam et al. found that EPHX1 and GSTP1 variants contribute to the occurrence of childhood asthma and increase asthma susceptibility to exposures from major roads based on the analysis for white children in Southern California [19]. Su et al. performed two-way and three-way gene-gene interactions to find the combining effect of GSTP1, INSIG2, and IL4Ra to lifetime asthma based on the Taiwan seventh-grade children [20]. Therefore, our results provide new evidences that COPD candidate genes may show interactive effects with lung-function-related traits. However, for 6MWT, MMRC, and BODE, the best interaction models only existed in SERPINE2 itself. Because COPD is a complex disease which is caused by the genetics contributions from multiple genes, therefore, genes derived from multiple gene-gene interactions may help reveal their cumulative effect effectively. SERPINE2 occurring in multiple gene-gene interactions has been approved for its association with COPD. In fact, many previous findings have confirmed that SERPINE2 gene polymorphisms are associated to COPD and may be involved particularly in the development of panlobular emphysema [21].

In addition, we know that the advantage of MDR is not only that it can deal with sparse and high-dimension data and therefore might uncover non-linear SNP-SNP interactions that are missed by QTL but also it can detect high-order interactions between genes which cannot be performed by QTL as the model complexity increases with the order of interactions. In the present study, we used GMDR and QMDR to detect the three-way interactions between genes and found that the best interactions were seen from some of these four genes. Furthermore, we have determined that the genes more frequently detected by GMDR, QMDR, and QTL were more likely to be functional, such as SERPINE2 [22]. This suggests that our results do not occur only by chance but might reveal some real biological links between genes. Especially, the interaction between EPHX1 and GSTP1 were approved by GeneMANIA web-based tool. The interactions detected by GMDR and QMDR seem more diverse and less influenced by the SNP main effects.

Moreover, we know that the most common variable selection strategy for interaction studies often select SNPs with main effects to test for the interactions. However, such an approach might cause the miss of some of the true interactions between genes. It has been reported that the false positive report probability (FPRP) [23] depends on prior probability that the association is real and the statistical power of the test, therefore selecting genes based on their functions can clearly reduce the FPRP [24]. In the present study, we select four genes: EPHX1, GSTP1, SERPINE2, and TGFB1 to explore their interaction effects contributing to COPD-related quantitative traits. In fact, our selected genes are based on the priori evidences that they are functionally important in the COPD development [13]. This selection may help reduce the FPRP. In the future, the further validation of the gene-gene interactions found in this study using other independent datasets will strengthen to confirm our results and provide further insight into the role of interacting genes in COPD etiology.

Furthermore, we must point out the limitations of the present study. Firstly, we limited our studies to interactions within four COPD-related genes, but interactions between genes based on the other priori biology evidences or on pathways may be related to disease risk as well and warrant further exploration. Secondly, a newly developed multivariate quantitative multifactor dimensionality reduction (Multi-QMDR) algorithm is approved to have better performance than QMDR and GMDR when multiple quantitative phenotypes are available [25]. This method summarized the multivariate phenotypes into a univariate score by dimensional reduction analysis, and then classify the samples accordingly into high-risk and low-risk groups. Although GMDR and QMDR are appropriate for analyzing the interactions of smaller number of biomarkers, Multi-QMDR will outperform these two methods when detecting the interactions between a greater number of biomarkers [25]. In our future work, Multi-QMDR will be used for exploring interaction effects between large numbers of variables including genetic and environment factors. Finally, a relatively small population was recruited and the relative small sample size might affect the results. After the patients were classified according to the genotype combinations, the size of the subgroups became small and this may also affect the statistical power [26]. Indeed, we can see that some best interactions were not significant after being adjusted by the permutation tests. Maybe added sample size can change this case. Therefore, our findings should be considered with caution. In the future, we will use a larger population and study more candidate genes taking into account the gene-gene or gene-environment interactions contributing to COPD phenotype or quantitative traits to elucidate the genetic pathogenesis of COPD.

## Methods

### Study population

We recruited 310 unrelated COPD patients aged 40–75 years from respiratory outpatient clinics at 12 hospitals in Beijing from October 2007 to March 2009. The cases are physician-diagnosed COPD; the pulmonary function test shows FEV_1_/FVC of less than 0.7 and FEV_1_%pre of less than 0.8 predicted and no evidence of primary asthma or other respiratory diseases. The control group comprised of 203 subjects with the same age range as the case group. They have no history of respiratory symptoms and exhibit normal pulmonary function of FEV_1_/FVC of more than 0.7 and FEV_1_%pre of more than 0.8 predicted. Written informed consent was obtained from every participating subject, and the study protocol was approved by the research ethics boards of all participating hospitals. The complete name of the ethics committee is Institutional Review Board of Beijing Chaoyang Hospital who approved this study. The detailed entry criteria and the baseline characteristics of study subjects were seen from our previous study [13, 27].

### Genotyping of SNPs

Genomic DNA was isolated from whole blood leukocytes by the conventional phenol-chloroform method. SNPs were genotyped using Illumina VeraCode technology performed on BeadXpress genotyping platform (Illumina Inc., USA). Forty-four tagging SNPs (MAF > 0.05) were genotyped to capture the common variants of the four genes (EPHX1, GSTP1, SERPINE2, and TGFB1) under pairwise mode with *r*^2^ threshold of 0.8. The detailed description was seen from our previous study [13, 27].

### COPD-related quantitative traits

In the present study, for COPD patients, we focused on seven COPD-related quantitative traits to perform our analysis. These traits were described simply as follows:

- *FEV*_*1*_*and FEV*_*1*_*%pre:* FEV_1_ is called the forced expiratory volume in one second, indicating the volume in a 1-s forced exhalation. It is then converted to a percentage of predicted based on your height, weight, and race normal named as *FEV*_*1*_*%pre*. FEV_1_%pre is a key value to be known by smokers and COPD patients in order to assess the severity of the disease [28]. In the present study, the FEV_1_%pre of COPD patients is less than 0.8.
- *FVC* is called the forced vital capacity, indicating the amount of air exhaled forcefully and quickly after maximum inspiration [29].
- *FEV*_*1*_*/FVC* is a calculated ratio used in the diagnosis of obstructive and restrictive lung disease. It represents the proportion of a person’s vital capacity that they are able to expire in the first second of expiration [30]. In the present study, the FEV_1_/FVC of COPD cases is less than 70 %.
- *6MWT* is the 6-min walk test which is the most commonly used exercise test in pulmonary rehabilitation. In the current study, the 6MWT was carried out according to the ATS guidelines [31]. Each patient was ordered to walk in a solid and flat corridor for 6 min as soon as possible. The test was repeated twice with an interval of at least 30 min. The best walking distances for two 6MWTs for each patient were recorded as the 6-min walking distance (6MWD).
- *MMRC* is the modified Medical Research Council dyspnea scale which uses a simple grading system to assess a patient’s level of dyspnea. The MMRC dyspnea scale classified the breathless into six grades (0 to 5) according to self-perceived breathlessness during daily activities [32]. Grade 5 represents the most severe category.
- *BODE* index is a multidimensional index comprising the BMI, degree of airway obstruction (FEV_1_%pre), functional dyspnea (MMRC dyspnea scale), and exercise capacity (6MWT). For the calculation of the BODE index, we used an empirical model as previously described [33]. For the first parameter, the value was 0 or 1. For the last three parameters, the patient received points ranging from 0 (lowest value) to 3 (highest value). The BODE index was the sum of points for each variable, ranging from 0 to 10. A higher BODE index score indicates a greater probability of patient mortality [34].

Considering that 6MWT, BODE, and MMRC are measure indexes only appropriate for COPD patients, therefore the quantitative traits measured for normal controls are four lung-function-related traits: FEV_1_, FEV_1_%pre, FVC, and FEV_1_/FVC, which were subjected to our further analysis.

### Statistical analysis

#### General characteristics of COPD-related quantitative traits for COPD patients

The general characteristics of COPD-related traits for patients are described, and the comparison among multiple genotype combination is performed using the analysis of variance (ANOVA) or Kruskal-Wallis test. A *P* value of <0.05 is considered statistically significant. All statistical analysis was completed using SPSS version 19.0 (SPSS Inc., Chicago, IL).

### Single marker analysis for COPD-related quantitative traits based on patients

For seven COPD-related quantitative traits for patients, we used the QTL method (general linear model) to perform single marker analysis using the PLINK software (http://pngu.mgh.harvard.edu/~purcell/plink/), and we used an additive model for gene effects. We included covariates age, sex, and pack-years of smoking in the model. A correction for multiple comparisons was performed using the Bonferroni procedure [35].

### Gene-gene interaction analysis for COPD-related quantitative traits

In the present study, we used recently developed GMDR and QMDR algorithms to test for the two-way and three-way gene-gene interactions with COPD-related quantitative traits based on the COPD patients and the whole samples, respectively. GMDR uses score-related statistics derived from a generalized linear regression model and allows covariates to be adjusted [11]. QMDR extends the MDR algorithm and works with quantitative or continuous phenotypes. Instead of comparing the case-control ratio of each multi-locus genotype to a fixed threshold, QMDR compares the mean value of each multi-locus genotype to the overall mean. The outcome between high- and low-level groups defined by the QMDR attribute is compared using a *T* test and then he *T* statistic is used as a training score to choose the best model [12]. In addition, we also used the QTL method (general linear model) to identify the two-way gene-gene interactions, and gene-gene pairs were considered significant if *P* value is smaller than 0.05. Those interactions with most significant *P* values were considered as the best two-way interaction models. The QTL was performed with the PLINK software (http://pngu.mgh.harvard.edu/~purcell/plink/). Considering that the covariates like age, sex, and smoking status might have a strong main effect and may potentially interfere with the ability of MDR and QTL to achieve their goal [35], we therefore included age, sex, and pack-years of smoking in these methods, respectively.

### Gene-gene interactions in the network

To extend/explore the potential joint genetic effects of these four genes (EPHX1, GSTP1, SERPINE2, and TGFB1) with other genes, we also used a web-based tool GeneMANIA [14] to find their interaction in any way (physically, genetically, etc.) in the network. Because GeneMANIA does not support pseudogenes, we thus manually queried the four genes: EPHX1, GSTP1, SERPINE2, and TGFB1, and used the automatic weighting for the network. For the network creation, we used only physical interactions, predicted interaction, pathways, and co-expression. GeneMANIA was accessed on 31 May 2013.

## Conclusion

In conclusion, our study suggests the potential interactions between EPHX1, SERPINE2, GSTP1, and TGFB1 contributing to COPD-related quantitative traits, such as FEV_1_, FEV_1_%pre and FVC. The MDR approaches used in this paper that have the potential in the identification of complex biological links contribute to COPD development processes. Our study provides further evidence for the genotype combinations at risk of developing COPD in Chinese Han population and improves understanding on the genetic etiology of COPD.

## Additional files

Additional file 1: Forty-four captured tagging SNPs involved in four genes (EPHX1, SERPINE2, GSTP1, and TGFB1) and their association with COPD phenotype using genotype-based chi-square tests. (DOC 103 kb) Additional file 2: The general characteristics of COPD-related quantitative traits for COPD patients (*n* = 310). (DOC 28 kb) Additional file 3: The description and comparison among multiple genotype combination contributing to seven COPD-related quantitative traits based on QMDR (*n* = 310 patients). (DOC 102 kb) Additional file 4: The best models identified by traditional QTL for COPD-related quantitative traits in Chinese Han population. (DOC 30 kb) Additional file 5: Gene-gene interaction models for COPD-related quantitative traits using traditional QTL method based on 310 patients. (PDF 138 kb)
