# Supplementary material for: Exploring the interaction among EPHX1, GSTP1, SERPINE2, and TGFB1 contributing to the quantitative traits of chronic obstructive pulmonary disease in Chinese Han population
Source: Hum Genomics. 2016 May 18;10:13. doi: 10.1186/s40246-016-0076-0 (PMC4870730; doi:10.1186/s40246-016-0076-0)
Supplement: Additional file 1: — Forty-four captured tagging SNPs involved in four genes (EPHX1, SERPINE2, GSTP1, and TGFB1) and their association with COPD phenotype using genotype-based chi-square tests. (DOC 103 kb) [file 40246_2016_76_MOESM1_ESM.doc]

**Additional file 1**. 44 captured tagging SNPs involved in four genes (EPHX1, SERPINE2, GSTP1 and TGFB1) and their association with COPD phenotype using genotype-based Chi-square tests

| Gene | SNP | Chromosome | Alleles | Region | Chi-square | *p*-value |
| --- | --- | --- | --- | --- | --- | --- |
| EPHX1 | rs1877724 | 1 | C/T | intronic | 0.1388 | 0.7095 |
| EPHX1 | rs1051740 | 1 | C/T | coding | 1.602 | 0.2056 |
| EPHX1 | rs1051741 | 1 | C/T | coding | 2.696 | 0.1006 |
| EPHX1 | rs2854450 | 1 | C/T | 5upstream | 0.04641 | 0.8294 |
| EPHX1 | rs2292558 | 1 | C/G | intronic | 0.01017 | 0.9197 |
| EPHX1 | rs2260863 | 1 | C/G | intronic | 2.826 | 0.09273 |
| EPHX1 | rs868966 | 1 | A/G | intronic | 0.4563 | 0.4993 |
| EPHX1 | rs1009668 | 1 | A/G | coding | 0.2504 | 0.6168 |
| EPHX1 | rs41266229 | 1 | A/G | intronic | 16.19 | 5.73E-05** |
| EPHX1 | rs2292568 | 1 | C/T | coding | 0.2384 | 0.6254 |
| EPHX1 | rs3766934 | 1 | G/T | intronic | 4.806 | 0.02837* |
| EPHX1 | rs3738040 | 1 | A/G | 5upstream | 0.6809 | 0.4093 |
| EPHX1 | rs2234922 | 1 | A/G | coding | 0.02807 | 0.8669 |
| SERPINE2 | rs6719480 | 2 | C/T | intronic | 1.283 | 0.2573 |
| SERPINE2 | rs4674841 | 2 | G/T | intronic | 1.445 | 0.2294 |
| SERPINE2 | rs17196253 | 2 | A/G | intronic | 2.569 | 0.1095 |
| SERPINE2 | rs975278 | 2 | A/G | intronic | 8.096 | 0.008054** |
| SERPINE2 | rs920251 | 2 | C/T | intronic | 1.551 | 0.3050 |
| SERPINE2 | rs6748795 | 2 | C/G | intronic | 3.406 | 0.06495 |
| SERPINE2 | rs3820766 | 2 | C/T | intronic | 7.607 | 0.005814** |
| SERPINE2 | rs10191694 | 2 | A/C | intronic | 2.278 | 0.2390 |
| SERPINE2 | rs7579646 | 2 | A/G | intronic | 2.232 | 0.1352 |
| SERPINE2 | rs13392495 | 2 | A/G | intronic | 4.518 | 0.03354* |
| SERPINE2 | rs282254 | 2 | C/T | intronic | 0.002744 | 0.9582 |
| SERPINE2 | rs7583463 | 2 | A/C | intronic | 1.429 | 0.193 |
| SERPINE2 | rs729631 | 2 | C/G | intronic | 12.1 | 0.000867** |
| SERPINE2 | rs6738983 | 2 | C/T | intronic | 0.03164 | 0.8588 |
| SERPINE2 | rs4674843 | 2 | A/G | intronic | 0.0715 | 0.3861 |
| SERPINE2 | rs861442 | 2 | A/G | intronic | 0.08717 | 0.5482 |
| SERPINE2 | rs7590948 | 2 | A/G | intronic | 1.384 | 0.3793 |
| SERPINE2 | rs2118409 | 2 | C/G | intronic | 0.952 | 0.6037 |
| SERPINE2 | rs6734100 | 2 | C/G | intronic | 6.516 | 0.01069* |
| SERPINE2 | rs6712954 | 2 | A/G | coding | 1.721 | 0.2016 |
| SERPINE2 | rs6736436 | 2 | C/T | 3downstream | 1.827 | 0.1765 |
| GSTP1 | rs4147581 | 11 | C/G | intronic | 0.2085 | 0.648 |
| GSTP1 | rs1138272 | 11 | C/T | coding | 7.971 | 0.00475** |
| GSTP1 | rs1695 | 11 | A/G | coding | 0.2837 | 0.5943 |
| GSTP1 | rs947895 | 11 | A/C | 3downstream | 0.003682 | 0.9516 |
| TGFB1 | rs6957 | 19 | A/G | 3utr | 1.003 | 0.3165 |
| TGFB1 | rs2241715 | 19 | G/T | intronic | 1.076 | 0.2995 |
| TGFB1 | rs12980942 | 19 | A/G | 3downstream | 0.05689 | 0.8115 |
| TGFB1 | rs2241718 | 19 | C/T | 3utr | 0.0009921 | 0.9749 |
| TGFB1 | rs2241713 | 19 | C/G | intronic | 3.112 | 0.0777 |
| TGFB1 | rs1800469 | 19 | C/T | 3downstream | 1.122 | 0.2894 |

**p*<0.05; ***p*<0.01
