# Supplementary material for: Exploring the interaction among EPHX1, GSTP1, SERPINE2, and TGFB1 contributing to the quantitative traits of chronic obstructive pulmonary disease in Chinese Han population
Source: Hum Genomics. 2016 May 18;10:13. doi: 10.1186/s40246-016-0076-0 (PMC4870730; doi:10.1186/s40246-016-0076-0)
Supplement: Additional file 2: — The general characteristics of COPD-related quantitative traits for COPD patients (n = 310). (DOC 28 kb) [file 40246_2016_76_MOESM2_ESM.doc]

**Additional file 2.** The general characteristics of COPD-related quantitative traits for COPD patients (n=310)

| Normalize variables | |  | Non-normalize variables | |
| --- | --- | --- | --- | --- |
| Quantitative traits | Mean±SD |  | Quantitative traits | Median (5th–95th percentile) |
| FEV_1_ | 1.27±0.38 |  | MMRC | 1 (0-4) |
| FEV_1_%pre | 45.70±11.89 |  | BODE | 2 (1-6) |
| FVC | 2.64±0.69 |  |  |  |
| FEV_1_/FVC(%) | 49.04±9.75 |  |  |  |
| 6MWT | 444.64±102.24 |  |  |  |
