# Supplementary material for: Exploring the interaction among EPHX1, GSTP1, SERPINE2, and TGFB1 contributing to the quantitative traits of chronic obstructive pulmonary disease in Chinese Han population
Source: Hum Genomics. 2016 May 18;10:13. doi: 10.1186/s40246-016-0076-0 (PMC4870730; doi:10.1186/s40246-016-0076-0)
Supplement: Additional file 3: — The description and comparison among multiple genotype combination contributing to seven COPD-related quantitative traits based on QMDR (n = 310 patients). (DOC 102 kb) [file 40246_2016_76_MOESM3_ESM.doc]

**Additional file 3.** The description and comparison among multiple genotype combination contributing to seven COPD-related quantitative traits based on QMDR (n=310 patients)

**①**FEV1

| **rs2292568*rs4147581** | **Mean±SD** | **F** | **P** |
| --- | --- | --- | --- |
| TT*GG | 1.30±0.40 | 2.084 | 0.045* |
| TT*CG | 1.29±0.41 |
| TT*CC | 1.18±0.39 |
| CT*GG | 1.08±0.29 |
| CT*CG | 1.25±0.26 |
| CT*CC | 1.25±0.18 |
| CC*GG | 1.82±0.22 |
| CC*CG | 1.32±0.54 |
| CC*CC | - |

*P<0.05

**②**FEV1%

| **rs1051741* rs6957** | **Mean±SD** | **F** | **P** |
| --- | --- | --- | --- |
| TT*GG | 47.47±13.71 | 3.228 | 0.046* |
| TT*AG | 44.10±9.45 |
| TT*AA | 47.29±13.31 |
| CT*GG | 41.16±14.31 |
| CT*AG | 35.57±10.01 |
| CT*AA | 59.92±12.15 |
| CC*GG | 49.00±11.79 |
| CC*AG | 43.64±14.35 |
| CC*AA | 45.49±12.01 |

*P<0.05

**③**FVC

| **rs7583463*rs2241713** | **Mean±SD** | **F** | **P** |
| --- | --- | --- | --- |
| CC*GG | 2.40±0.59 | 3.226 | 0.046* |
| CC*CG | 2.57±0.74 |
| CC*CC | 2.96±0.87 |
| AC*GG | 2.76±0.74 |
| AC*CG | 2.75±0.67 |
| AC*CC | 2.98±0.73 |
| AA*GG | 2.56±0.51 |
| AA*CG | 3.18±0.74 |
| AA*CC | 2.73±0.79 |

*P<0.05

**④FEV1/FVC (%)**

| **rs17196253*rs6748795** | **Mean±SD** | **F** | **P** |
| --- | --- | --- | --- |
| GG*GG | 48.26±10.10 | 0.595 | 0.781 |
| GG*CG | 50.18±10.78 |
| GG*CC | 48.57±6.25 |
| AG*GG | 50.30±9.17 |
| AG*CG | 48.81±9.10 |
| AG*CC | 46.98±8.21 |
| AA*GG | 44.00±7.25 |
| AA*CG | 52.33±11.37 |
| AA*CC | 35.98±12.01 |

**⑤BODE**

| **rs4674841*rs6748795** | **Median(Quartile interval)** | **Chi-square** | **P** |
| --- | --- | --- | --- |
| TT*GG | 2 (2) | 16.237 | 0.023* |
| TT*CG | 3 (2) |
| TT*CC | 3 (2) |
| GT*GG | 2 (3) |
| GT*CG | 2 (2) |
| GT*CC | 2 (2) |
| GG*GG | 3 (1) |
| GG*CG | 3 (2.5) |
| GG*CC | - |

*P<0.05

**⑥ MMRC**

| **rs2118409*rs6712954** | **Median(Quartile interval)** | **Chi-square** | **P** |
| --- | --- | --- | --- |
| GG*GG | 1 (1) | 11.995 | 0.151 |
| GG*AG | 1 (2) |
| GG*AA | 1 (1.5) |
| CG*GG | 1 (1) |
| CG*AG | 1 (1) |
| CG*AA | 1 (0) |
| CC*GG | 1 (1) |
| CC*AG | 1 (1.5) |
| CC*AA | 1 (2) |

**⑦** 6MWT

| **rs7583463*rs2118409** | **Mean±SD** | **F** | **P** |
| --- | --- | --- | --- |
| CC*GG | 467.38±55.34 | 2.711 | 0.007** |
| CC*CG | 428.66±115.83 |
| CC*CC | 434.05±120.18 |
| AC*GG | 400.64±132.24 |
| AC*CG | 472.31±72.42 |
| AC*CC | 461.65±94.98 |
| AA*GG | 417.50±80.85 |
| AA*CG | 486.94±64.25 |
| AA*CC | 465.17±75.48 |

**P<0.01
