# Supplementary material for: Exploring the interaction among EPHX1, GSTP1, SERPINE2, and TGFB1 contributing to the quantitative traits of chronic obstructive pulmonary disease in Chinese Han population
Source: Hum Genomics. 2016 May 18;10:13. doi: 10.1186/s40246-016-0076-0 (PMC4870730; doi:10.1186/s40246-016-0076-0)
Supplement: Additional file 4: — The best models identified by traditional QTL for COPD-related quantitative traits in Chinese Han population. (DOC 30 kb) [file 40246_2016_76_MOESM4_ESM.doc]

**Additional file 4.** The best models identified by traditional QTL for COPD-related quantitative traits in Chinese Han population

| COPD-related quantitative traits | The best two-way interaction models  (n=310 patients) | The best two-way interaction models  (n=310 patients+ 203controls) |
| --- | --- | --- |
| FEV_1_ | EPHX1(rs3738040)*GSTP1(rs4147581) | EPHX1(rs3766934)*GSTP1(rs947895) |
| FEV_1_%pre | EPHX1(rs1051740)*TGFB1(rs12980942) | GSTP1(rs1695)*TGFB1(rs2241713) |
| FVC | SERPINE2(rs13392495)*TGFB1(rs2241713) | SERPINE2(rs975278)*TGFB1(rs1800469) |
| FEV_1_/FVC (%) | SERPINE2(rs6738983)*SERPINE2(rs7583463) | SERPINE2(rs6748795)*TGFB1(rs2241713) |
| BODE | SERPINE2(rs10191694)*SERPINE2(rs7583463) | _ |
| MMRC | SERPINE2(rs975278)*TGFB1(rs2241718) | _ |
| 6MWT | EPHX1(rs1051741)*SERPINE2(rs7583463) | _ |
