# Supplementary material for: Exploring the interaction among EPHX1, GSTP1, SERPINE2, and TGFB1 contributing to the quantitative traits of chronic obstructive pulmonary disease in Chinese Han population
Source: Hum Genomics. 2016 May 18;10:13. doi: 10.1186/s40246-016-0076-0 (PMC4870730; doi:10.1186/s40246-016-0076-0)
Supplement: Additional file 5: — Gene-gene interaction models for COPD-related quantitative traits using traditional QTL method based on 310 patients. (PDF 138 kb) [file 40246_2016_76_MOESM5_ESM.pdf]

A

		EPHX1 (rs3738040)		
		GG	AG	AA
GSTP1 (rs4147581)	GG			
	CG			
	CC			1.53

FEV1

B

		EPHX1 (rs1051740)		
		TT	CT	CC
TGFB1 (rs12980942)	GG			
	AG			
	AA			50.39

FEV1%pre

C

		SERPINE2 (rs13392495)		
		GG	AG	AA
TGFB1 (rs2241713)	GG			
	CG			
	CC			3.63

FVC

D

		SERPINE2 (rs6738983)		
		TT	CT	CC
SERPINE2 (rs7583463)	CC	55.18		
	AC			
	AA			

FEV1/FVC (%)

E

		SERPINE2 (rs10191694)		
		CC	AC	AA
SERPINE2 (rs7583463)	CC			
	AC			3.36
	AA			

BODE

F

		SERPINE2 (rs975278)		
		GG	AG	AA
TGFB1 (rs2241718)	TT			1.87
	CT			
	CC			

MMRC

G

		EPHX1 (rs1051741)		
		TT	CT	CC
SERPINE2 (rs7583463)	CC			
	AC			
	AA	470.16		

6MWT

Additional file 5: Gene-gene interaction models for COPD-related quantitative traits using traditional QTL method based on 310 patients. (**A**) FEV_1; (**B**) FEV_1%pre; (**C**) FVC; (**D**) FEV_1/FVC(%); (**E**) BODE; (**F**) MMRC; (**G**) 6MWT. For FEV_1, FEV_1%pre, FVC, FEV_1/FVC(%) and 6MWT, the red shaded cells indicate the maximum of mean of all genotype combinations respectively. For BODE and MMRC, the green shaded cells indicate the maximum of median of all genotype combinations respectively.
